# The dual impact of social media: evolving beauty perceptions and cosmetic procedure practices among patients and providers

**DOI:** 10.25122/jml-2024-0390

**Published:** 2024-12

**Authors:** Jumana Hussain Timraz, Rayyan Rafat Samman, Syeda Nafeesa Hashim, Saleha Khan, Maya Faissal Alhomieed, Lara Osama Al Hartany, Laura Mashtoub, Arwa Sindi

**Affiliations:** 1 General Medicine Practice Program, Batterjee Medical College, Jeddah, Saudi Arabia; 2 Plastic Surgery Department, The First Clinic, Jeddah, Saudi Arabia

**Keywords:** cosmetic procedures, social media, plastic surgery, Saudi Arabia

## Abstract

Social media is gaining popularity in Saudi Arabia, influencing the concept of beauty and cosmetic surgical needs, particularly among younger individuals. This study aimed to understand how social media is changing the face of cosmetic surgery by reflecting new beauty standards. A comprehensive literature review was conducted, focusing on studies published between 2015 and 2024 from databases such as PubMed and Scopus, examining the impact of social media on decisions related to plastic surgery. Our study accessed multiple studies revealing a concerning trend in the influence of social networks, especially on appearance-related decisions. Some persuasive appeals include false images, celebrity endorsements, and the use of pictures before or after the reconstruction of cosmetic surgery. Additionally, marketing strategies employed by plastic surgeons were found to contribute to the rising demand for both surgical and minimally invasive procedures. This article provides a detailed understanding of how social media can influence ideals and trends in cosmetic surgery while also highlighting the psychological impacts of impossible standards of beauty as well as the ethical implications of advertising practices in the industry. It also examines whether the influence of social media primarily serves the financial interests of providers or adds pressure on them to meet patients’ heightened expectations. The limitations in prior research highlight the need for studies involving diverse populations and a closer examination of the potential long-term effects of social media on cosmetic surgery trends and perceptions of beauty.

## INTRODUCTION

Social media has profoundly reshaped many aspects of modern life, significantly altering perceptions of beauty and influencing decision-making processes. This impact is even more pronounced in Saudi Arabia due to the interplay between rich cultural traditions and the country’s progressive modernization. Between 2020 and 2024, platforms such as Instagram, Snapchat, and TikTok have rapidly grown, reshaping beauty standards, public perceptions, and their demand for cosmetic procedures. This effect is particularly evident among younger demographics, who are highly active on these platforms and, consequently, more susceptible to their influence. Saudi youth primarily rely on social media for information on esthetics and beauty trends, with influencers and celebrities playing a crucial role in promoting beauty standards and cosmetic surgery [1-4]. These platforms serve as a perfect venue to broadcast images and videos normalizing and increasing the popularity of plastic surgery. Research shows that frequent exposure to such content affects body image perceptions, heightening the desire for cosmetic procedures [2].

The cultural context of Saudi Arabia plays a crucial role in understanding the impact of social media on cosmetic surgery. Historically, cosmetic surgery in the country was met with skepticism, frequently associated with religious and cultural concerns underlining modesty and restraint. However, through the increasing transparency of esthetic procedures on social media, perceptions started to change [3]. Nowadays, cosmetic procedures are more widely perceived as pathways to personal fulfilment and social acceptance, reflecting a broader cultural shift toward appearance and self-expression. This perceptual change underlines the role of social media in reframing social norms related to beauty in Saudi Arabia.

From a psychological perspective, social media exerts a significant influence on body image, creating and perpetuating unrealistic beauty standards. The constant exposure to edited and filtered images fosters a culture of comparison and inadequacy. In Saudi Arabia, these pressures are intense among young adults, the most active social media users. This environment has contributed to a rise in body image dissatisfaction, driving many to seek cosmetic interventions in pursuit of the unattainable beauty ideals propagated on social media. This idealized chase comes with great psychological burdens, such as anxiety and lowered self-esteem, which further propels individuals to seek cosmetic interventions [2].

Plastic surgeons in Saudi Arabia have also adapted to this transition, promoting their services through social media. Recent research indicates a substantial rise in invasive and non-invasive procedures driven by digital beauty trends. Procedures such as rhinoplasties, lip fillers, and body sculpting have become increasingly popular to conform to social media-driven beauty ideals [2]. Many practitioners actively engage with audiences through social media, sharing before-and-after photos, procedural details, and patient testimonials. This direct interaction helps educate potential clients about different procedures and builds trust and understanding. As a result, social media has become a powerful instrument for plastic surgeons to reach and attract patients, playing a decisive role in their decision-making process [3].

We aimed to provide a comprehensive understanding of how social media is reshaping the landscape of cosmetic surgery in Saudi Arabia, highlighting the evolving dynamics of beauty standards and consumer behavior in the modern era.

## MATERIAL AND METHODS

### Literature search

A comprehensive and systematic literature search was conducted to identify relevant studies published between 2020 and 2024 that explored the relationship between social media usage and individuals' decisions to undergo plastic surgery. The search was performed using PubMed and Google Scholar using specific keywords: 'social media', 'plastic surgeon', 'cosmetic surgery', 'influence', and 'Saudi Arabia'. To minimize the risk of omitting relevant studies, the reference lists of the identified articles were also manually reviewed for additional sources.

### Data extraction

Variables included authors, year of publication, study design, sample size, and the primary outcomes related to the impact of social media on plastic surgery decisions. Detailed notes were taken on the methodologies used in each study, including how social media exposure was measured, the specific aspects of plastic surgery decisions that were analyzed, and any confounding variables controlled for in the analysis. A summary of the key characteristics of the included studies is presented in Table 1.

**Table 1:** Summary of included studies examining the influence of social media on individuals' decisions to undergo cosmetic procedures

Study	Type of study	Sample size	Age (years)	% of people influenced by social media	Key findings
Taishan *et al*., 2024 [4]	Cross-sectional	568	20 to 80 years	42.6%	The two primary factors influencing participants to consider plastic surgery were surgeons’ advertisements on social media and before-and-after photos.
AlBahlal *et al*., 2023 [5]	Cross-sectional	2249	>12 to >51 years	43.3 %	Approximately 35.9% of participants were influenced by surgeons’ ads on social media. Participants aged 21–30 were most affected by social media when considering cosmetic surgery or other cosmetic procedures.
Obeid *et al*., 2022 [6]	Cross-sectional	205	18 to >35 years	52.7 %	The most significant factor influencing participants to undergo rhinoplasty was the before-and-after photos posted on social media, with Snapchat being the most frequently used platform.
Suwayyid *et al*., 2020 [7]	Cross-sectional	911	>18 to >45 years	38.6%	Among the participants, 13% had a history of plastic surgery, and 38.6% intended to undergo surgery. Factors significantly associated with undergoing plastic surgery included gender, age, income, social media images, and appearance motivations (P < 0.05).
Badi Aldosari, 2020 [8]	Cross-sectional	653	>18 to 65 years	37.8%	Social media was used by 98.3% of participants, and 93.4% frequently took selfies, which motivated them to undergo cosmetic procedures. The sample included 25.1% men and 74.9% women.
Alhusaini *et al*., 2022 [9]	Cross-sectional	1064	Anyone above 18 years	27%	The most common age group was < 25 years, with more women than men. Decisions to undergo plastic surgery were influenced by social media influencers (79%), before-and-after photos (37.1%), and social media ads (27%).
Arab *et al*., 2019 [10]	Cross-sectional	375	18–30 years old	48.5%	48.5% of respondents were influenced by advertisements for cosmetic treatments. Of those, two-thirds considered nonsurgical options (n = 280, 70.7%), while only 18.7% were interested in surgical procedures. About 22.4% (n = 183) considered future procedures due to social media influence, and 14.6% (n = 119) had previously undergone treatment.
Sindi *et al*., 2023 [11]	Cross-sectional	385	18 to over 50 years	Frequent influence - I22.5% Constant influence - 6.1%	The study found that 10.8% of participants had undergone cosmetic procedures. For facial procedures, anti-wrinkle injections scored the highest mean (2.1), followed by rhinoplasty (1.9). Women rated anti-wrinkle injections highest (2.4), while men favored rhinoplasty (1.4). In body procedures, liposuction scored 2.1 and abdominoplasty 1.9, with women rating them higher than men.

## RESULTS

The comprehensive literature review conducted for this study revealed significant findings regarding the influence of social media on cosmetic surgery decisions among Saudi patients and providers. The analysis included multiple studies published between 2020 and 2024, highlighting trends in cosmetic procedures, psychological impacts, and cultural shifts in beauty perceptions. A total of five relevant studies were identified and analyzed.

Taishan *et al*. [4] surveyed 568 participants aged 20 to 80 years, with 42.6% reporting that social media influenced their decisions to consider plastic surgery. The most impactful factors were advertisements and before-and-after photos shared on social platforms [4]. Similarly, AlBahlal *et al*. [5] surveyed 2,249 individuals over 12 and found that 43.3% were influenced by surgeons’ advertisements on social media [5]. Obeid *et al*. [6] focused on 205 participants aged 18 to over 35 and found that 52.7% were influenced by social media, with before-and-after photos being a primary motivator for seeking rhinoplasty, especially on platforms like Snapchat [6]. In a separate study by Suwayyid *et al*. [7], which included 911 participants aged 18 to 45 years, 38.6% reported being influenced by social media, with various factors, including gender and income, impacting their decisions [7]. Additionally, in the study by Aldosari *et al*. [8], which included 653 participants aged 18 to 65, 37.8% were influenced by social media, with 98.3% using these platforms, indicating the significant role of selfies in driving cosmetic surgery decisions [8]. The study of Alhusaini *et al*. [9] involved 1,064 participants above 18 years and revealed that 27% of them were influenced by social media. The most common group affected were women under 25 years, of whom 79% decided to undergo plastic surgery influenced by social media. Additionally, 37% were affected by before-and-after images of procedures, and 27% were influenced by cosmetic surgery adverts on social media [9]. Moreover, a study by Arab *et al*. [10] investigating the influence of social media revealed that 48.5% of participants were influenced by social media, with Snapchat being identified as the most impactful platform. Interestingly, the study also found that 22.8% of participants preferred consulting famous plastic surgeons promoted on social media. Lastly, Sindi *et al*. [11] reported that women were more inclined to consider cosmetic surgery than men. Participants with lower self-esteem were particularly prone to contemplating aesthetic procedures.

Across all studies, the percentage of participants influenced by social media in their decisions to pursue cosmetic surgery ranged from 27% to 52.7%. This indicates a strong link between social media exposure and the desire for surgical interventions. Platforms like Snapchat and Instagram emerged as particularly impactful, with respondents often citing specific content, such as before-and-after photos and influencer endorsements, as pivotal in shaping their perceptions of beauty and surgical options. Younger individuals, especially those aged 20 to 30, showed a marked susceptibility to social media influences. The literature highlighted that this demographic is more likely to consider cosmetic enhancements due to exposure to idealized beauty standards prevalent on social media. Furthermore, the studies collectively point to a concerning trend where social media exposure leads to increased body dissatisfaction and low self-esteem. Participants frequently reported feelings of inadequacy and pressure to conform to unrealistic beauty ideals, which, in turn, prompted considerations for cosmetic surgery as a potential solution.

**Figure 1:**
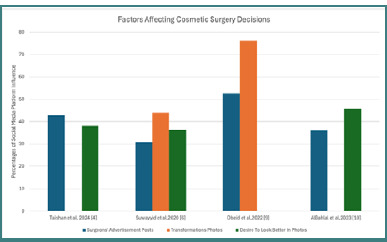
Common factors affecting surgery decisions across our findings

Lastly, the studies indicated a gradual shift in cultural attitudes toward cosmetic surgery in Saudi Arabia. Traditionally viewed with skepticism, cosmetic procedures are increasingly accepted as pathways to self-expression and personal fulfillment, particularly among younger generations influenced by social media. In summary, the results of this literature review highlight the significant role social media plays in influencing cosmetic surgery decisions among Saudi patients. The findings reveal a strong relationship between social media exposure and the desire for cosmetic enhancements, underscored by psychological impacts and evolving cultural norms, as seen in Figure 1. This emphasizes the need for further research and consideration of ethical implications in cosmetic surgery marketing and patient care.

## DISCUSSION

In recent years, plastic surgery has become increasingly popular in Saudi Arabia, with a notable rise in the number of procedures performed each year. For instance, in 2020, Saudi Arabia recorded 24,964 plastic surgery operations, ranking 22nd among the top 25 nations for cosmetic procedures [1]. Moreover, over half of Saudi women (55.4%) opted for cosmetic procedures, highlighting a significant increase in the number of women seeking surgical enhancements [12]. This trend indicates that cosmetic surgery is becoming closely associated with contemporary beauty standards, particularly among women.

Our study aimed to provide a thorough understanding of how social media is transforming the cosmetic surgery landscape in Saudi Arabia. We identified recent studies that reveal the profound effect of social media on the perceptions of beauty and the practices surrounding cosmetic surgery among patients and providers in the region. This review indicates a growing body of evidence showing that social media plays a crucial role in influencing people's decisions to pursue cosmetic procedures, especially among younger audiences. Various studies report that 37.8% to 52.7% of participants were influenced by social media when considering cosmetic surgery [6,8]. Platforms such as Snapchat and Instagram are particularly influential, with many respondents citing specific content, including before-and-after photos and influencer endorsements, as key factors shaping their beauty perceptions. Younger individuals, particularly those aged 20 to 30, are especially vulnerable to these social media influences. This demographic is more inclined to contemplate cosmetic enhancements due to the exposure to idealized beauty standards on these platforms. Furthermore, the studies collectively indicate a troubling trend where increased social media exposure correlates with heightened body dissatisfaction and lower self-esteem. Participants often expressed feelings of inadequacy and pressure to conform to unrealistic beauty ideals, prompting them to consider cosmetic surgery as a possible solution [10,12].

Many individuals attribute their desire for cosmetic surgery to the influence of media and celebrity culture [1,9,10-12]. This finding is consistent with previous research emphasizing how media portrayals and cultural beauty ideals shape people's perceptions of their bodies and their aspirations for cosmetic procedures. It suggests that images, media messages, and celebrity endorsements can significantly influence individuals' views on attractiveness and motivate them to consider surgical interventions. Additionally, social media promotes unattainable beauty standards, which drives demand for cosmetic procedures [11,13]. Research by Mavis *et al*. suggests that social media negatively impacts individuals' self-esteem regarding body image and self-perception [14]. Multiple studies highlight how platforms like Instagram and Snapchat have normalized the pursuit of ideal beauty, with users frequently exposed to curated images that set unrealistic standards. Similarly, Taishan *et al*. [4] reveal that 42.6% of participants were influenced by social media ads and before-and-after images when contemplating plastic surgery [4]. Such exposure fosters a culture where cosmetic enhancements are sought after and expected, particularly among youth who are the most active users of these platforms [1].

The psychological consequences of social media engagement are significant. Studies indicate a link between frequent social media usage and issues such as body dissatisfaction, anxiety, and low self-esteem. This is especially evident among young adults in Saudi Arabia, who often compare their appearances to the idealized images they encounter online. The literature underscores that this culture of comparison can lead to an increased desire for cosmetic procedures as individuals strive to meet these unattainable standards. Research by Obeid *et al*. supports this notion, noting that 52.7% of participants reported that before-and-after photos influenced their decisions [6].

Furthermore, our review highlights how plastic surgeons have adapted their marketing strategies to effectively utilize social media. Surgeons now engage with potential clients through these platforms, showcasing their work and building trust through transparency. This aligns with findings from AlBahlal *et al*., who revealed that 43.3% of participants were influenced by social media advertisements from surgeons [5]. The direct interactions facilitated by social media educate potential clients and increase the visibility of cosmetic procedures, driving demand for surgical and non-invasive options. Cultural factors in Saudi Arabia significantly shape these trends. Traditionally, cosmetic surgery faced skepticism due to cultural and religious values prioritizing modesty. However, the literature indicates a shift in perceptions, with cosmetic procedures increasingly viewed as avenues for self-expression and personal fulfilment [5]. This change is supported by the findings of Suwayyid *et al*., who identified key factors influencing plastic surgery decisions, including exposure to social media and cosmetic television programs [7]. The evolving attitudes towards beauty and self-enhancement reflect broader societal changes, suggesting that social media is reshaping the norms around cosmetic surgery in Saudi Arabia.

Internationally, social media has also seen similar trends in cosmetic surgery. Various studies have demonstrated the impact of social media platforms on plastic surgery patients considering cosmetic procedures. One study found that 95% of patients exploring cosmetic surgery consulted online sources, including social media, before deciding [15]. A cross-sectional study conducted in the United States investigating the preferred social media platforms and engagement patterns revealed an average respondent age of 44 years, with a predominance of female participants, similar to findings in KSA. Facebook emerged as the most engaging platform, followed by Instagram, while Snapchat, Twitter, and Pinterest demonstrated lower engagement levels. This contrasts with the trends in Saudi Arabia, where Snapchat was identified as a highly influential platform, as highlighted by the studies discussed above [16].

Interestingly, Walden *et al*. discovered that while more than half of patients use Google or other online resources for information, only about 10% directly consult a plastic surgeon [17]. The American Society of Plastic Surgeons conducted a survey in 2021, revealing that nearly half of all patients considering plastic surgery credited their decision to social media influence, similar to findings in KSA [18]. Another study exploring the link between young adults’ perceptions of cosmetic procedures and their social media use found that higher engagement with visually focused platforms like Instagram and TikTok was associated with an increased intention to pursue cosmetic enhancements, suggesting a reciprocal relationship [19].

Additionally, another study found that most (95%) of patients used the Internet as a primary source of information before consultation, with 68% relying on it as their first search method. Social media influenced 46% of patients, and 40% of those reported feeling strongly impacted when choosing a specific surgeon. Moreover, 85% of plastic surgeons expressed concern that information from social media could create unrealistic expectations, reflecting broader trends of body dissatisfaction and lower self-esteem [20].

### The impact of social media and the rising trend of male patients and plastic surgery in Saudi Arabia

The impact of social media on men seeking cosmetic treatments and plastic surgeries in Saudi Arabia has been widely studied in the past years due to the rising trends of these procedures. Similar to its influence on women, social media content shared by famous artists, bodybuilders, and cosmetologists plays a substantial role in shaping men’s body dissatisfaction. Furthermore, the increased accessibility of such content, facilitated by celebrities’ widespread presence on digital platforms, has normalized cosmetic procedures among men, making them more socially acceptable. By sharing their surgical experiences and results, influencers and public figures encourage their audiences to explore these trends, fostering an environment where cosmetic enhancements are perceived as viable tools for boosting self-esteem and embracing broader cultural acceptance of such changes [21]. In addition, these advertisements have contributed to a cultural shift in Saudi Arabia, driving the growth of cosmetology clinics and the cosmetic surgery market. Healthcare professionals are tailoring their marketing strategies to address the specific needs of male patients in the region. They provide targeted consultations designed exclusively for men, aiming to normalize and support their decisions to pursue cosmetic enhancements. This targeted approach has significantly influenced men’s perceptions of beauty, leading to a growing interest in liposuction, hair transplantation, rhinoplasty, and skin rejuvenation treatments [22]. As a result, social media platforms have positively influenced the cosmetic market in Saudi Arabia, particularly among men, by encouraging and normalizing cosmetic procedures that were previously considered contrary to traditional norms. However, research indicates that women remain more likely than men to be influenced by social media in their decisions to pursue cosmetic enhancements [23,24].

### Ethical consideration

The increasing use of social media by plastic surgeons to promote their services and attract potential patients raises several critical ethical considerations. These considerations revolve around autonomy, beneficence and nonmaleficence, and justice [24]. Beyond merely obtaining consent, patients must be thoroughly informed about the implications of sharing their images or videos online and the potential consequences. Patients must acknowledge, through a consent form, that once such materials are posted online, they may not be entirely removable and may cease to remain the sole property of the surgeon [24]. Patients must not be coerced into consenting, and the surgeon should stress that this decision would not affect patient care [25]. Surgeons and residents must be aware of their unintentional impact on patients’ choices. Patients are likely to trust the decisions made by their surgeons; hence, consideration must be given to sponsorship and reward for medical brand promotion. Therefore, to ensure authenticity, medical professionals should only advertise products they truly think are the best available. They should also be willing to disclose that the content is a sponsored or paid advertisement by using certain metadata tags such as 'ad' or 'paid advertisement' [24].

When using social media, a delicate balance exists between maintaining patient privacy and providing content that may be perceived as entertaining or engaging. According to the principle of nonmaleficence, the obligation to "do no harm" to a patient is paramount. Harm can take many forms, including physical malpractice or the unethical use of patient information, which could jeopardize their well-being. For instance, patients may not fully understand that relinquishing their anonymity to allow their cases to be shared could result in their information being reused across multiple platforms. Additionally, images and videos are difficult to remove once posted online, even at the patient’s request. In addition, any posted material must be reviewed to ensure the patient's experience is not underplayed or sexualized [24,25].

According to the principle of justice, medical resources must be distributed equally. Medical justice in plastic surgery and social media is best defined as fairness in obtaining top ranking through pay-for-play social media recognition. The non-digital doctor-patient relationship upon which medicine was founded must be authenticated. Patients should be informed if their doctor pays to be listed as a "top doctor" rather than receiving unsolicited recognition for their work. Only with such transparency can social media be utilized effectively while still honoring the obligations of a medical professional to their patients. As plastic surgeons are professional practitioners with great responsibility towards both their profession and their patients, they must uphold the highest standards of ethical behavior. In conclusion, as challenging as it may be, it is beneficial for the esthetic plastic surgery specialty community to maintain professionalism in their social media activities and apply ethical guidelines locally and internationally [24-26].

### Future implications

Advancements in technology, changing social norms, and patient safety continue to shape the development of plastic surgery [27]. Practitioners should prioritize the integration of cutting-edge digital technologies, such as 3D imaging and augmented reality, to enhance pre-operative planning and patient outcomes. Additionally, a growing emphasis on minimally invasive procedures aligns with patient preferences for safer and less complex interventions. Open communication about the risks and realistic outcomes of procedures is increasingly important in the context of growing emphasis on moral behavior, particularly in relation to the influence of social media on body image. In addition, to maintain current knowledge of advancements in regenerative medicine, including stem cell therapies, and to guarantee that the greatest patient care and safety guidelines are followed, practitioners should participate in ongoing education [28]. Lastly, future research should focus on exploring the long-term effects of social media on cosmetic surgery trends and body image perceptions.

## CONCLUSION

Social media plays a significant role in plastic surgery in Saudi Arabia, influencing demand for cosmetic procedures and reshaping the interaction between patient and physician by enhancing involvement and information distribution. However, it is important that plastic surgeons uphold their ethical standards by ensuring that patients receive proper care and realistic expectations. Social media has a lasting impact on how people perceive themselves in terms of beauty and body image. Despite this, people continue to use social media as a platform for inspiration and to gain knowledge about plastic surgery.
